# RN-D^3^: detection, differentiation, and delineation of radiation necrosis on post-contrast MRI

**DOI:** 10.3389/frai.2026.1891883

**Published:** 2026-08-06

**Authors:** Patrick Salome, Nicolò Cogno, Hoyeon Lee, Gregory Buti, Keyur D. Shah, Felix Ehret, Amy Jordan Wisdom, Helen A. Shih, Harald Paganetti, Ibrahim Chamseddine

**Affiliations:** 1 Department of Radiation Oncology, Mass General Brigham Cancer Institute and Harvard Medical School, Boston, MA, United States; 2 Clinical Cooperation Unit Translational Radiation Oncology, German Cancer Research Center (DKFZ), Heidelberg, Germany; 3 Department of Diagnostic Radiology and Centre of Cancer Medicine, LKS Faculty of Medicine, University of Hong Kong, Hong Kong, Hong Kong SAR, China; 4 Department of Radiation Oncology and Winship Cancer Institute, Emory University, Atlanta, Georgia; 5 Department of Radiation Oncology, Charité—Universitätsmedizin Berlin, Freie Universität Berlin and Humboldt-Universität zu Berlin, Berlin, Germany; 6 German Cancer Consortium (DKTK), Partner Site Berlin, a Partnership Between DKFZ and Charité – Universitätsmedizin Berlin, Berlin, Germany

**Keywords:** deep learning segmentation, external validation, foundation models, post-contrast MRI, radiation necrosis (RN)

## Abstract

**Purpose:**

Radiation necrosis (RN) is an important complication of radiation therapy (RT) and is challenging to assess radiographically because lesions are prone to inconsistent delineation, often necessitating intracranial surgery to obtain a diagnosis. This study evaluated how established deep learning segmentation models can be retrained on an institutional RN cohort, assessed external generalization, and integrated the best components into RN-D3, an end-to-end pipeline for RN detection, differentiation, and delineation.

**Methods and materials:**

We trained models on a cohort of 52 patients with RN after proton beam RT treated at Massachusetts General Hospital (2004–2016) and tested these models on an external cohort of 29 patients with 39 RN lesions from the MOLAB brain metastasis dataset (photon-based RT, five Spanish institutions, 2005–2021). Six architectures were evaluated: four convolutional neural networks (nnU-Net, STU-Net, TRIAD PlainConv, and Spark3D) and two transformers (SwinUNETR and TRIAD SwinB). Training strategies included training from scratch, supervised pretraining, self-supervised pretraining, and foundation model initialization. Matched-encoder pairs enabled direct comparison of pretraining effects. Performance was evaluated by Dice similarity coefficient (DSC), surface Dice at 2 mm, and lesion detection rate. A post-processing pipeline combining encoder features and shape radiomics was assessed for reducing false positives. The best segmentation model and this classifier were combined into RN-D^3^, an end-to-end pipeline evaluated on 115 brain metastasis (BM) lesions from MOLAB for RN-versus-non-RN differentiation.

**Results:**

Spark3D and STU-Net achieved the highest external DSC (median 0.69 [IQR 0.50–0.84] and 0.65 [0.29–0.88]), followed by baseline nnU-Net (0.61 [0.39–0.83]). For Spark3D and STU-Net, large lesions (≥1 mL, *n* = 21) were detected at 100% (median DSC 0.77 and 0.73) and small lesions (<1 mL, *n* = 18) at 78 and 67% (DSC 0.60 and 0.58). The remaining models detected 17–22% of small lesions with a median DSC near zero. False-positive filtering removed 2/13 and 7/14 false positives for Spark3D and STU-Net, retaining all true detections for Spark3D and 85% for STU-Net. RN-D^3^ achieved an end-to-end sensitivity of 0.90 and a specificity of 0.88 on 115 BM lesions for RN-versus-non-RN differentiation. The two transformer-based architectures evaluated showed larger drops from internal to external cohorts (ΔDSC −0.36 to −0.42 vs. − 0.12 to −0.20 for CNNs), though all architectures degraded externally.

**Conclusion:**

RN-D^3^ integrates a CNN segmentation backbone with an RN-versus-non-RN classifier into an end-to-end pipeline addressing detection, differentiation, and delineation of RN. On external data, RN-D^3^ achieved high end-to-end sensitivity and high specificity in patients with brain metastases, with detection of small lesions the main determinant of missed cases. Small-lesion detection remains the primary translational bottleneck.

## 1 Introduction

Radiation necrosis (RN), a late treatment-related effect, has become increasingly clinically important (Lawrence et al., 2010). RN appears as a contrast-enhancing lesion on conventional MRI and presents two central diagnostic challenges: distinguishing RN from artifactual radiographic changes or from true tumor progression or recurrence, which require different clinical management (Ali et al., 2019; Vellayappan et al., 2018; Chamseddine et al., 2024). Because physician assessments of imaging are often inadequate for diagnosis, patients may undergo craniotomy to obtain tissue for histopathologic confirmation. Radiographic diagnosis is an urgent need, but manual RN delineation, required for reproducible volumetric monitoring, is time-consuming, subject to inter-observer variability, and poorly scalable for routine clinical follow-up. Automated segmentation could enable scalable monitoring and facilitate downstream analyses (Cao et al., 2023; Salari et al., 2023; Sadique et al., 2024). However, RN segmentation is challenging due to heterogeneous lesion appearance and variable sizes, both of which affect model generalization.

To date, deep learning (DL) studies have segmented related but distinct targets, including radiographic changes (Ho et al., 2025), progression, necrosis, and edema together (Haskell-Mendoza et al., 2024), and RN-versus-progression differentiation (Ressa et al., 2025), with reported Dice similarity coefficient (DSC) values of 0.72–0.85 on single-institution cohorts. However, none have addressed RN as a primary, isolated segmentation target, evaluated transformer-based or large-scale pretrained architectures for this task, or reported external validation on independent datasets. Critically, none evaluated performance on small RN lesions, despite early RN frequently presenting as sub-centimeter foci where early detection is most valuable. Medical image segmentation has progressed from CNN-based encoders (Ronneberger et al., 2015) to transformer-augmented architectures (Hatamizadeh et al., 2022) that capture global contextual information, and to large-scale pretrained and foundation models that reduce dependence on expensive task-specific annotations (Ma et al., 2024a; Zhou et al., 2021).

However, real-world clinical RN datasets remain limited in size, constraining the application of deep learning methods that require large training cohorts. Foundation models and large-scale pretrained encoders may offer a path to learn meaningful representations of such rare clinical entities under realistic data constraints. In this study, we asked how well established deep learning segmentation models can be adapted to RN using a moderate-sized institutional cohort, a setting that reflects the data availability of many clinical centers. We evaluated automated RN identification on post-contrast MRI through detection, differentiation, and delineation, comparing performance on small and large lesions and on an independent external cohort to reflect clinical variability. The study was designed to examine how model architecture and training strategy influence generalization under these conditions, including matched-encoder comparisons that isolate the effect of pretraining from architectural differences. To verify that the trained models learned RN-specific representations rather than generic radiographic-change detection, we additionally evaluated specificity on brain metastasis lesions from the same external cohort. We then combined the best-performing components into RN-D^3^, an end-to-end pipeline addressing detection, differentiation, and delineation.

## 2 Materials and methods

**Figure 1:**
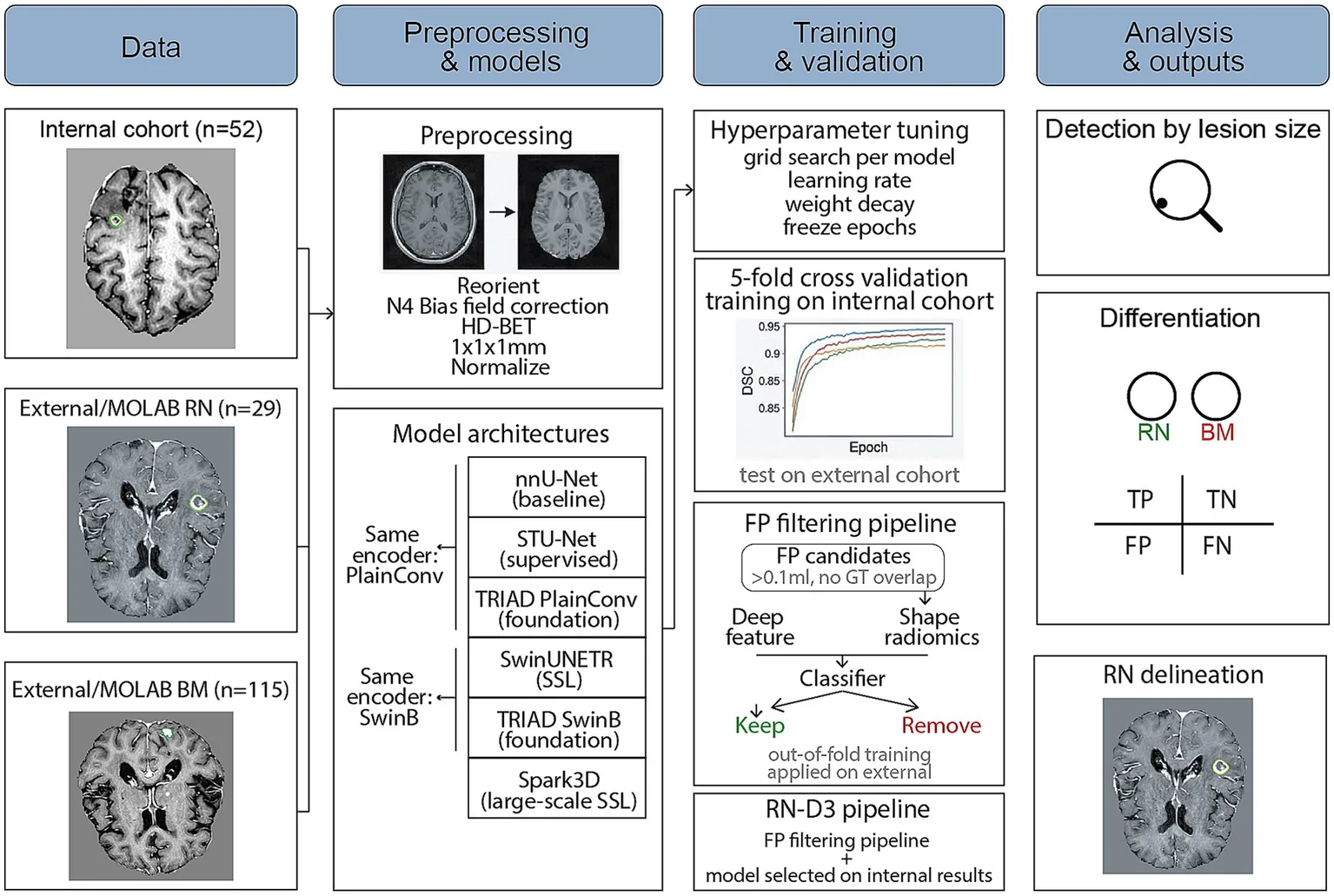
Study design overview. The pipeline includes two cohorts: an internal cohort (*n* = 52, proton beam radiotherapy [RT]) and an external MOLAB cohort (photon-based RT), with two evaluation subsets: 39 RN lesions and 115 brain metastasis lesions. Both are processed through a shared preprocessing pipeline and evaluated across six convolutional neural network (CNN) and transformer architectures with varying pretraining strategies, grouped by shared encoder. Models were trained by 5-fold cross-validation on the internal cohort; the external test set was used for final evaluation only. Performance was assessed using Dice similarity coefficient (DSC), surface Dice at 2 mm, and detection rate, stratified by lesion size (<1 mL vs. ≥ 1 mL), with an additional post-processing pipeline to reduce false positives and improve specificity against brain metastases. Candidate lesions and embeddings for the false-positive (FP) classifier were generated out-of-fold: each patient’s candidates were produced by the segmentation model that excluded that patient, so classifier training data were independent of segmentation training.

We evaluated established deep learning architectures and post-processing strategies, addressing detection, differentiation, and delineation through RN-D3, an end-to-end pipeline combining a segmentation backbone with a false-positive classifier (see Figure 1).

### 2.1 Datasets

This study included two retrospective cohorts: an internal cohort used for model development and an independent external test cohort. The study populations were derived from larger clinical cohorts to reflect the real-world incidence of radiation-induced effects. For both cohorts, only the first post-treatment MRI time point at which a contrast-enhancing lesion consistent with RN was identified was included per patient, resulting in one scan per patient. Lesions were stratified at a 1 mL threshold into small (<1 mL) and large (≥1 mL) subgroups. Patient and imaging characteristics are summarized in Supplementary Table S1.

The internal cohort included 52 patients with RN identified through a screening of 165 patients treated with proton beam RT at Massachusetts General Hospital (Boston, MA) between 2004 and 2016. Treated tumors comprised intracranial and skull-base primary malignancies. This screening population includes a subset of 130 patients previously analyzed for patient heterogeneity in RN development (Niyazi et al., 2020; Niemierko et al., 2021). RN was identified by the treating physician, graded according to CTCAE v4.03, and determined using a documented multi-factor reference standard comprising neuroradiologist interpretation, independent review of each case by two neuro-oncology specialists, and serial-imaging evolution; 7 of 52 cases were confirmed histologically at surgery, and the remaining 45 were diagnosed by adjudicated clinical–radiographic follow-up over a median of 45 months (range 13.9–110), with stabilization or resolution of the contrast-enhancing lesion and without subsequent progression or lesion-directed antitumor therapy. Radiation-necrosis regions were contoured on post-contrast T1-weighted MRI in patients in whom a necrosis region could be delineated (Niyazi et al., 2020; Niemierko et al., 2021); these contours served as the segmentation labels for model training. Median age was 53.2 years (range 19–82); 29 were male and 23 female. Median prescribed dose was 68.3 Gy(RBE) (range 59.4–80.5) in a median of 35 fractions (range 30–40). Median time from RT to RN identification was 18 months (IQR: 12–31.5). MRI was acquired on 13 scanner models across three manufacturers (Siemens, Philips, GE); median slice thickness was 4.0 mm (range 1.0–5.0). Of the 52 lesions, 21 (40%) were <1 mL and 31 (60%) were ≥1 mL.

The external test cohort included 29 patients with RN and 39 RN lesions, drawn from a total of 75 unique patients in the publicly available MOLAB brain metastasis (BM) dataset and collected at five Spanish institutions between 2005 and 2021 (Ocaña-Tienda et al., 2023). Twenty patients received SRS alone (median 20.0 Gy, range 16.0–24.0, in a single fraction), and 9 received SRS combined with WBRT (total dose median 30.0 Gy, range 16.5–75.0, in 3–15 fractions). Median age was 59.0 years (range 27–73); 15 were male and 14 female. Median time from RT to RN identification was 14.0 months (IQR 10.4–19.3; *n* = 26). The median number of RN lesions per patient was 1 (IQR 1–2; range 1–4); 21/29 patients had one lesion, 7/29 had two lesions, and 1/29 had four lesions. MRI was acquired on 5 scanner models across three manufacturers; median slice thickness was 1.3 mm (range 1.0–2.0). Of the 39 lesions, 18 (46%) were <1 mL and 21 (54%) were ≥1 mL. Lesion masks were generated using semi-automatic segmentation with manual correction and reviewed by a board-certified radiologist, with RN labels established by the dataset authors using longitudinal growth-dynamics assessment (Ocaña-Tienda et al., 2023). For the differentiation analysis, an additional 115 BM lesions, from 59 unique patients in the same dataset, were included as a specificity cohort (median 1 lesion per patient, IQR 1–2, range 1–7); 79/115 (69%) were <1 mL and 36/115 (31%) were ≥1 mL.

### 2.2 Image preprocessing

Post-contrast T1-weighted sequences were identified and verified using MR-Class (Salome et al., 2023a). Before model training, images were reoriented to RAS coordinate space, followed by N4 bias field correction to address MRI field inhomogeneities. Brain extraction was then performed using HD-BET (Isensee et al., 2019). Images were resampled to an isotropic voxel spacing of 1 mm^3^ using B-spline interpolation; lesion masks were resampled using nearest-neighbor interpolation. Intensity normalization was applied as:

$z=(\text{clip}(x,p_{0.1},p_{99.9})-\mu )/\sigma$ Where x is the raw voxel intensity, p₀.₁ and p₉₉.₉ are the 0.1st and 99.9th percentiles of the brain voxel distribution, and *μ* and *σ* are the mean and standard deviation of the clipped intensities.

### 2.3 Model architectures

Six architectures were evaluated, including CNN- and transformer-based architectures, representing three pretraining approaches: supervised pretraining, self-supervised masked autoencoder (MAE) pretraining, and foundation model initialization. CNNs extract features through local convolutional operations, scanning the image region by region with fixed-size kernels. In contrast, transformers consider the entire image at once to capture global context through an attention mechanism that weights the relevance of each image region relative to all others. All segmentation models followed a U-Net-style encoder–decoder design, in which an encoder progressively downsamples the image into hierarchical feature representations and a symmetric decoder with skip connections upsamples them back to a voxel-wise segmentation map; the models differed only in their encoder backbone and initialization, while the decoder was identical across all models, isolating the effect of the encoder and its pretraining. nnU-Net, a self-configuring CNN framework that has become the standard baseline for medical image segmentation, served as the CNN baseline trained from scratch using a residual encoder backbone (30.4 M parameters) (Isensee et al., 2021). The remaining five models were initialized with pretrained weights: STU-Net shares the same residual encoder as nnU-Net but was initialized with supervised pretraining on TotalSegmentator (Huang et al., 2023; Wasserthal et al., 2023); TRIAD PlainConv uses the same architecture, initialized with foundation model weights pretrained on 131,170 MRI volumes (Yang et al., 2025). SwinUNETR and TRIAD SwinB share an identical Swin Transformer encoder (39.8 M parameters), initialized with self-supervised MAE pretraining (Hatamizadeh et al., 2022) and TRIAD foundation model weights (Yang et al., 2025), respectively. Spark3D uses a substantially larger residual encoder (106.7 M parameters total; 90.3 M encoder) pretrained via MAE on 39,000 brain MRI volumes (Wald et al., 2025). All models were fine-tuned end-to-end. Architecture details, including per-model encoder depth, parameter counts, and pretraining sources, are summarized in Supplementary Table S2.

### 2.4 Model training

All models were trained using 5-fold cross-validation (CV) on the internal cohort, with patient-level splits to prevent data leakage. Training ran for up to 300 epochs with early stopping when validation DSC did not improve for 50 consecutive epochs. Patch size (112 × 160 × 160 voxels) and batch size (Ali et al., 2019) were fixed across models. For each architecture, learning rate, weight decay, encoder learning rate multiplier, and encoder freeze duration were tuned once by grid search using 3-fold patient-level CV on the internal cohort; the selected hyperparameters were then held fixed for the 5-fold CV used for model comparison. Training used the AdamW optimizer with a base learning rate (base_LR) applied to the decoder and head, and an encoder learning rate of base_LR × multiplier.

The training objective combined Dice loss and binary cross-entropy (BCE), both computed within the brain mask, as:

L=L_Dice+L_BCE where ℒ_Dice = 1 − (2*Σ* ŷy)/(Σ ŷ + Σ y + *ε*) is computed only over samples with ≥50 foreground voxels (volume-aware masking), and ℒ_BCE = −[y log ŷ + (1 − y) log(1 − ŷ)] is applied unconditionally within the brain mask; ŷ denotes the predicted probability, y the binary ground truth RN mask (defined per section 2.1), and *ε* a smoothing term. Volume-aware masking prevents Dice from being dominated by background-only patches in the highly imbalanced RN segmentation setting.

Data augmentation was applied on-the-fly during training and comprised independent random flips along all three axes, random affine transformations (rotation up to ±19° per axis, scaling 0.9–1.1), 3D elastic deformation, MR-style intensity augmentations (bias field, gamma, Gaussian noise, and contrast adjustment), coarse dropout, and thick-slice simulation along the z-axis. Transforms were applied independently per sample with probabilities between 0.17 and 0.50.

### 2.5 RN-versus-non-RN classifier pipeline

We developed a binary classifier to distinguish RN from non-RN among predicted lesion candidates (connected components in the binary segmentation mask, ≥0.1 mL) output by the segmentation models, with the goal of reducing false positives while preserving lesion sensitivity. Candidates that overlapped the ground-truth RN were labeled as RN; those without overlap were labeled as non-RN. Candidates and their embeddings were extracted from internal out-of-fold predictions: for each of the five CV folds, the segmentation model was applied only to the patients held out of that fold, and the resulting candidates and embeddings were concatenated across folds. Consequently, every patient’s candidates and features were generated by a model not trained on that patient, so the classifier training data were independent of segmentation training at the patient level. For each candidate, the encoder produced a variable-length set of voxel-level latent vectors covering the candidate’s spatial extent; these were mean-pooled (a permutation- and size-invariant aggregation) into a single 320-dimensional latent representation. Shape-based radiomic features (8 features: volume, surface area, sphericity, elongation, flatness, major and minor axis length, and maximum three-dimensional diameter) were computed using PyRadiomics (van Griethuysen et al., 2017). Three feature configurations were evaluated in the ablation study: deep features only, shape radiomics only, and their combination (Deep+Rad, 328 dimensions). For each configuration, both logistic regression and XGBoost classifiers were assessed. Threshold selection used patient-level 5-fold CV to optimize sensitivity-constrained F1, so that no patient’s candidates appeared in both training and validation folds; the trained classifier and selected threshold were then evaluated on external prediction.

### 2.6 RN-D^3^: pipeline assembly and differentiation analysis

We assembled RN-D^3^ by combining the segmentation model with the highest internal detection rate and DSC together with the sensitivity-constrained logistic-regression classifier from Section 2.5. To evaluate whether RN-D^3^ captured RN-specific patterns rather than generic post-contrast enhancement, we performed lesion-level RN-versus-non-RN differentiation (with BM as the non-RN class) on the external cohort. Of 39 RN lesions, 35 were flagged by RN-D3 after classifier filtering; the remaining 4 lesions were retained and counted as false negatives so that differentiation metrics reflect end-to-end performance. All 115 BM lesions were included. Inference settings, model weights, and the classifier threshold (selected via internal cross-validation per Section 2.5) were fixed based on the RN experiments; no tuning was performed on the external BM cohort. The RN-D^3^ configuration was selected using internal results only; the external MOLAB cohort was used solely for final evaluation and contributed to neither training, threshold selection, nor model selection. A lesion (RN or BM) was considered “flagged as RN” if at least one predicted candidate overlapped it after classifier filtering. Predicted candidates overlapping neither annotated RN nor BM lesions were reported separately as additional detections and not included in the confusion matrix. A predefined subgroup analysis was performed in patients with both RN and BM lesions. We note that the classifier was trained to distinguish RN from non-RN candidates (where non-RN candidates were segmentation false positives, not BM specifically); BM rejection therefore reflects generalization of this RN-versus-non-RN decision rather than a classifier explicitly trained on RN-versus-BM.

### 2.7 Evaluation and statistical analysis

Model performance was evaluated on the external test cohort using three metrics: DSC as the primary segmentation accuracy measure; Surface Dice at 2 mm tolerance, which quantifies boundary agreement by measuring the proportion of predicted and ground truth surface points within 2 mm of each other; and lesion detection rate, defined as the proportion of lesions for which the Euclidean distance between the ground truth and predicted centroid was ≤10 mm. False positive (FP) lesions were defined as predicted connected components (26-connectivity) with no overlap with any ground truth lesion and a volume of at least 0.1 mL. Metrics were stratified by ground truth lesion volume (<1 mL vs. ≥ 1 mL). The generalization gap was quantified descriptively as ΔDSC (external DSC − internal CV mean DSC). Continuous metrics (DSC, Surface Dice) are reported as median [IQR] pooled across all cases; detection rate is reported as a proportion with 95% Wilson confidence intervals. For the differentiation analysis, sensitivity, specificity, positive predictive value (PPV), negative predictive value (NPV), and accuracy are reported with two-sided 95% Wilson score confidence intervals. Differentiation metrics were computed at both the lesion level and the patient level; for the patient-level analysis (using the MOLAB patient identifier), a patient was RN-positive if they had at least one RN lesion and was flagged positive if any of their lesions was flagged as RN, with BM-only patients forming the negative class. Metrics were computed end-to-end, so that RN lesions missed at the detection stage, or patients whose only RN lesion was missed, were counted as false negatives. Uncertainty in median DSC was quantified using a patient-clustered nonparametric bootstrap (10,000 resamples), and the top-ranked models were compared using the Wilcoxon signed-rank test on paired per-lesion DSC, with Holm correction for multiple comparisons. Paired model comparisons on the external cohort used Wilcoxon signed-rank tests for per-lesion DSC, patient-clustered bootstrap 95% confidence intervals for median DSC differences, and McNemar tests for paired detection status, with Holm correction across tests.

### 2.8 Implementation details

All models were implemented in PyTorch 2.7.1 and trained on three NVIDIA RTX A5500 GPUs. All code and trained model weights are publicly available at: https://github.com/chamseddine-lab/rn-d3.

## 3 Results

### 3.1 Segmentation performance and generalization

Detection and delineation performance were assessed using detection rate, DSC, and Surface Dice at 2 mm. On internal CV, all models achieved median DSC of 0.79–0.87: Spark3D (0.87 [IQR 0.79–0.91]), STU-Net (0.85 [0.79–0.89]), TRIAD SwinB (0.84 [0.72–0.89]), TRIAD PlainConv (0.84 [0.68–0.87]), nnU-Net (0.79 [0.63–0.86]), and SwinUNETR (0.79 [0.57–0.86]). In the external test cohort, Spark3D and STU-Net ranked highest (median DSC 0.69 [0.50–0.84] and 0.65 [0.29–0.88]), followed by nnU-Net (0.61 [0.39–0.83]). Spark3D achieved the highest median DSC in 68% of bootstrap resamples and STU-Net in 31%, but no pairwise difference among the three leading models was significant in either DSC or detection (Spark3D vs. STU-Net: Wilcoxon *p* = 0.36; STU-Net vs. nnU-Net: McNemar Holm-adjusted *p* = 1.000; all DSC-difference confidence intervals spanning zero, all adjusted *p* ≥ 0.12). The best CNN outperformed the best transformer (Spark3D vs. TRIAD SwinB: paired median DSC difference 0.17, 95% CI 0.01 to 0.53, Wilcoxon *p* < 0.001; detection-rate difference 35.9 percentage points, 95% CI 20.5 to 53.8), and a family-level analysis confirmed this gap (pooled median DSC 0.61 vs. 0.02; cluster-robust mean difference 0.22, *p* < 0.001; detection-rate difference 24.4 percentage points, 95% CI 13.9 to 36.8). TRIAD PlainConv (0.33 [0.00–0.73]), TRIAD SwinB (0.06 [0.00–0.68]), and SwinUNETR (0.02 [0.00–0.64]) showed lower external performance, likely reflecting the mismatch between the TRIAD pretraining domain and the RN segmentation task. Surface Dice at 2 mm followed the same ranking. Full results are presented in Table 1.

**Table 1:** Segmentation performance on internal CV and external test cohorts.

Model	Internal DSC median [IQR] (95% CI)	External DSC median [IQR] (95% CI)	Internal surface dice at 2 mm median [IQR]	External surface dice at 2 mm median [IQR]	Internal detection (%)	External detection (%)
Spark3D	0.87 [0.79–0.91] (0.83–0.88)	0.69 [0.50–0.84] (0.56–0.82)	0.94 [0.88–0.98]	0.83 [0.67–0.94]	90.4	87.2
STU-Net	0.85 [0.79–0.89] (0.82–0.87)	0.65 [0.29–0.88] (0.58–0.85)	0.91 [0.84–0.95]	0.74 [0.61–0.86]	88.5	82.1
nnU-Net (baseline)	0.79 [0.63–0.86] (0.70–0.83)	0.61 [0.39–0.83] (0.50–0.75)	0.81 [0.77–0.89]	0.54 [0.45–0.74]	75.0	84.6
TRIAD PlainConv	0.84 [0.68–0.87] (0.79–0.85)	0.33 [0.00–0.73] (0.00–0.54)	0.91 [0.86–0.93]	0.14 [0.04–0.62]	82.7	52.6
TRIAD SwinB	0.84 [0.72–0.89] (0.80–0.87)	0.06 [0.00–0.68] (0.00–0.38)	0.93 [0.84–0.96]	0.15 [0.03–0.75]	82.7	51.3
Swin-UNETR	0.79 [0.57–0.86] (0.72–0.81)	0.02 [0.00–0.64] (0.00–0.43)	0.80 [0.62–0.93]	0.04 [0.00–0.56]	73.1	53.8

Dice similarity coefficient (DSC) reported as median [interquartile range, IQR] (95% CI; patient-clustered bootstrap); surface dice at 2 mm as median [interquartile range, IQR]. Detection rate is the proportion of ground-truth lesions detected. Models sorted by external DSC. The three leading models did not differ significantly in external DSC or detection (all Holm-adjusted *p* ≥ 0.12); CNNs significantly outperformed transformers (family-level *p* < 0.001).

**Figure 2:**
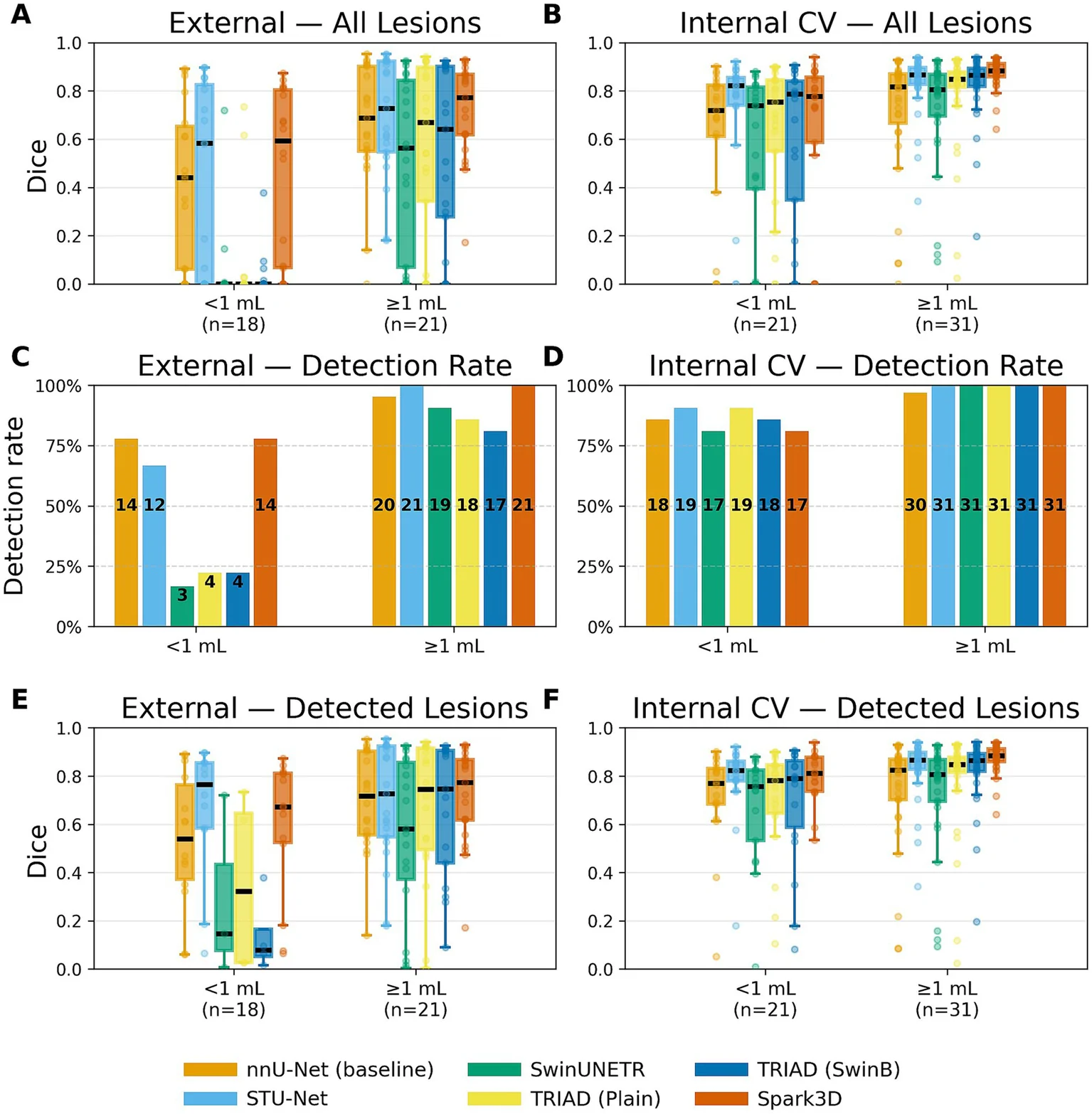
Segmentation performance stratified by lesion size and cohort. **(A)** External dice similarity coefficient (DSC) by lesion size (<1 mL, *n* = 18; ≥1 mL, *n* = 21). **(B)** Internal cross-validation (CV) DSC by lesion size (<1 mL, *n* = 21; ≥1 mL, *n* = 31). **(C)** External lesion detection rate; numbers within bars indicate detected/total. **(D)** Internal CV detection rate. **(E)** External DSC is restricted to detected lesions. **(F)** Internal CV DSC restricted to detected lesions. Boxes show median and interquartile range (IQR); whiskers extend to 1.5 × IQR. Dashed line at DSC = 0.5. The leading models did not differ significantly (all Holm-adjusted *p* ≥ 0.12); CNNs significantly outperformed transformers (family-level *p* < 0.001).

DSC varied by lesion size. Externally, for lesions ≥1 mL, Spark3D and STU-Net detected all 21 lesions (100%) with median DSC of 0.77 and 0.73, with detection ranging from 81 to 100% and median DSC from 0.56 to 0.77 across all models. For lesions <1 mL, Spark3D and STU-Net achieved the highest median DSC (0.60 and 0.58), with 78 and 67% detection, respectively, while SwinUNETR, TRIAD PlainConv, and TRIAD SwinB had detection of 17–22% (Figure 2).

**Figure 3:**
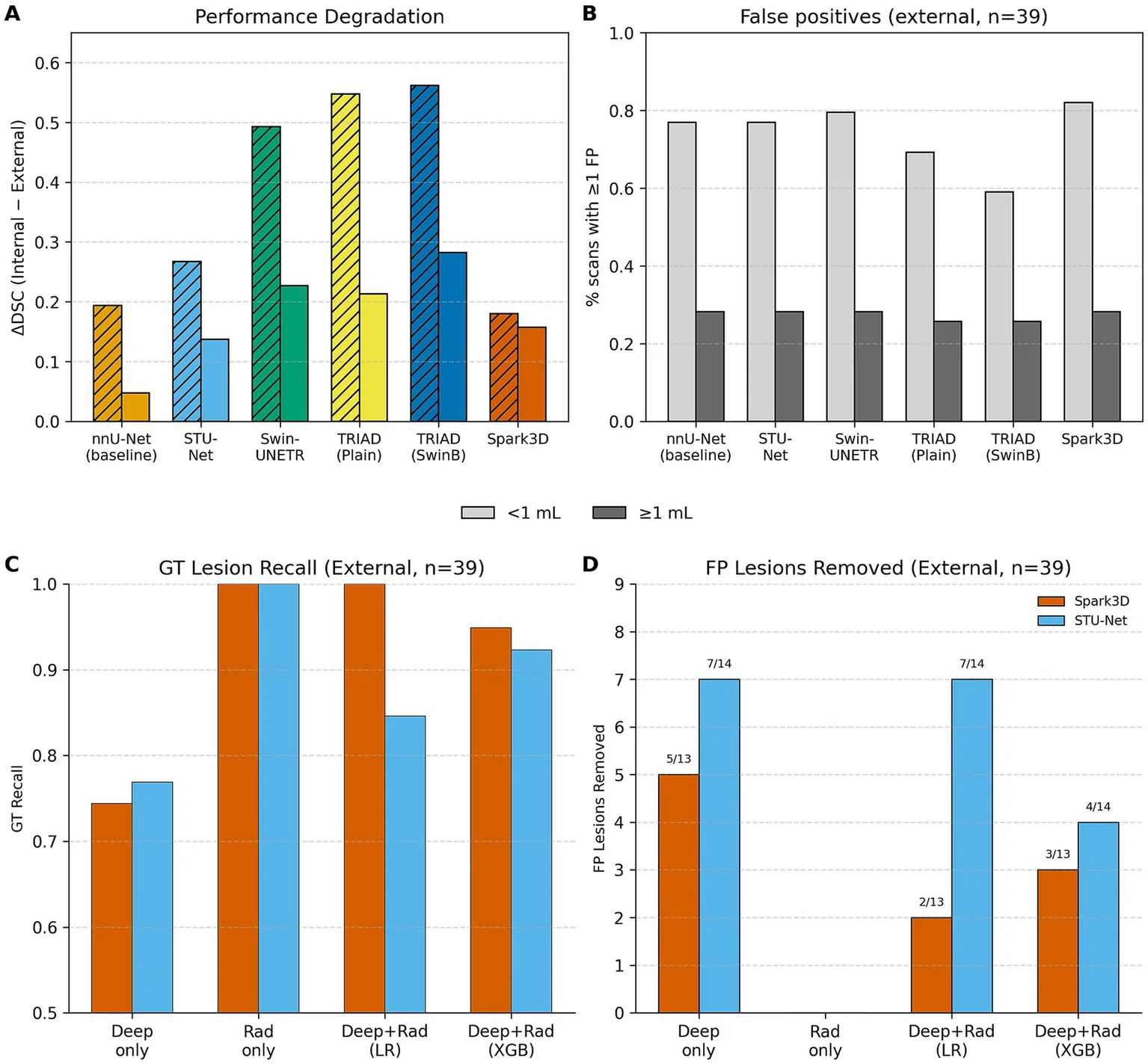
Generalization and false positives. **(A)** ΔDSC (external − internal mean dice similarity coefficient [DSC]) per model, stratified by lesion size. Hatched bars: <1 mL; Solid: ≥1 mL. **(B)** Proportion of external scans with ≥1 false-positive (FP) component (≥0.1 mL), by lesion size. **(C)** Ground-truth lesion sensitivity for four FP classifier configurations (deep features alone, shape radiomics alone, and their combinations with logistic regression or XGBoost). **(D)** FP components removed per configuration; Fractions indicate removed/total available.

Generalization gap (ΔDSC, external minus internal mean DSC) was smallest for nnU-Net (−0.12), Spark3D (−0.18), and STU-Net (−0.20), and largest for TRIAD SwinB (−0.42), TRIAD PlainConv (−0.38), and SwinUNETR (−0.36; Figure 3A). False positive burden was highest for Spark3D, nnU-Net, and STU-Net (82, 77, and 77% of scans with ≥1 FP for small lesions; Figure 3B).

### 3.2 Architecture versus pretraining

Within the PlainConv encoder group, STU-Net showed a non-significant advantage over nnU-Net externally (median DSC 0.65 vs. 0.61; McNemar detection *p* = 1.000, DSC difference 95% CI 0.00 to 0.04). At the same time, TRIAD PlainConv had external median DSC 0.33 (ΔDSC −0.38). Within the SwinB encoder group, SwinUNETR and TRIAD SwinB performed similarly externally (median DSC 0.02 vs. 0.06). Spark3D (ResEnc-L, 90.3 M encoder parameters) achieved the highest external median DSC overall.

### 3.3 RN-versus-non-RN classifier

**Figure 4:**
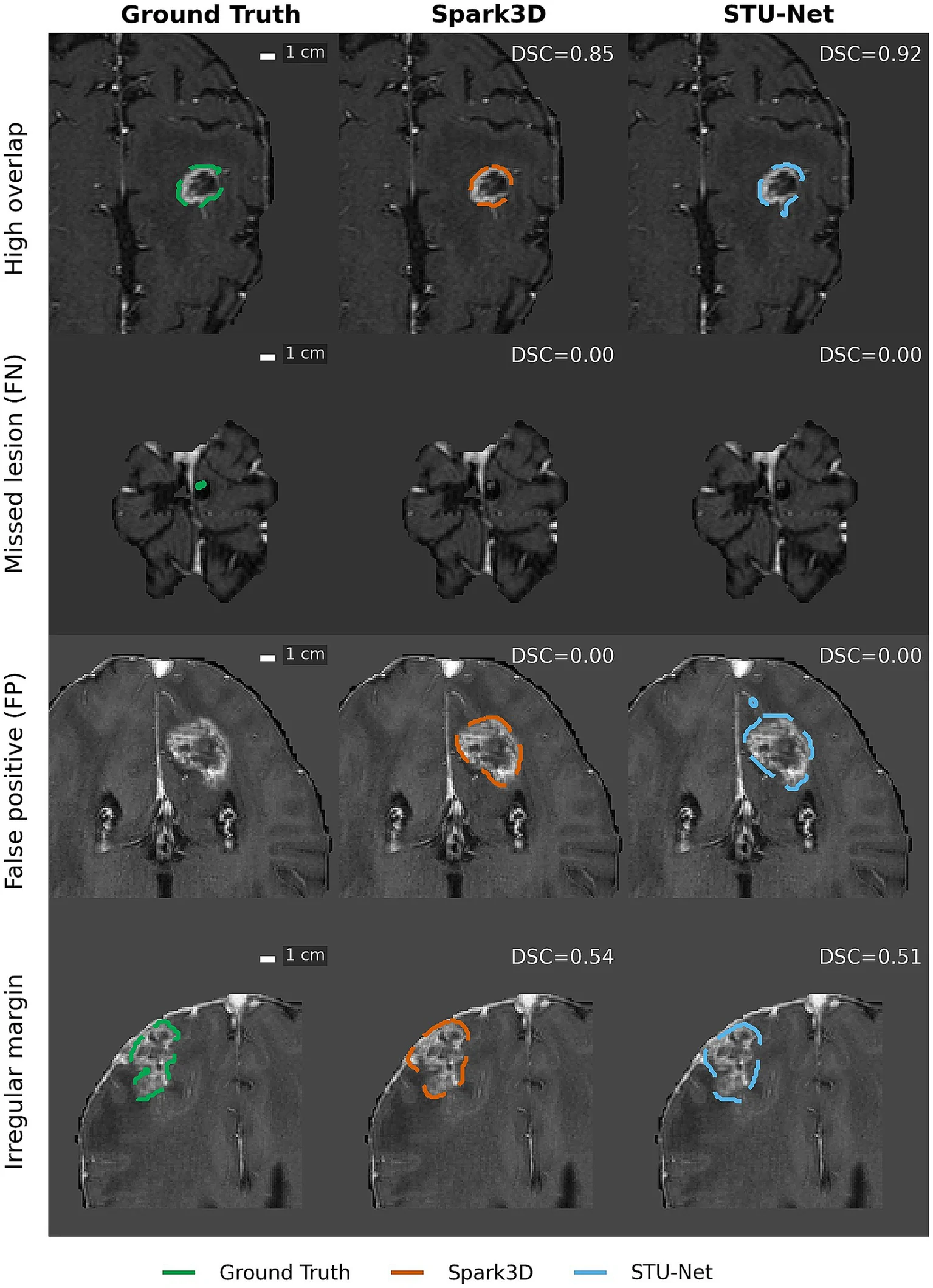
Representative segmentation examples on the external cohort. Ground truth (green), Spark3D (orange), and STU-Net (blue) contours on post-contrast T1-weighted MRI. Four scenarios are shown, with Dice similarity coefficient (DSC) values per model: high-overlap segmentation (Spark3D DSC = 0.85, STU-Net DSC = 0.92), missed lesion (both DSC = 0.00), false positive without ground-truth enhancement (both DSC = 0.00), and irregular-margin lesion (Spark3D DSC = 0.54, STU-Net DSC = 0.51). Scale bar = 1 cm.

The classifier was trained on 2,303 lesion candidates (92 RN, 2,211 non-RN) extracted from Spark3D internal out-of-fold predictions. We evaluated the four FP filter configurations described in Section 2.5. On the external cohort, Spark3D produced 13 FP components, and STU-Net produced 14. Deep features alone achieved ground-truth (GT) sensitivity of 0.74 for Spark3D and 0.77 for STU-Net, removing 5/13 and 7/14 FP, respectively, but at the cost of missed lesions. Shape radiomics alone retained all true detections for both models, but removed no FP. The combined Deep + Rad logistic regression classifier with sensitivity-constrained thresholding retained all true detections for Spark3D (2/13 FP removed) and 85% for STU-Net (7/14 FP removed), representing the best balance of FP reduction and sensitivity preservation (Figures 3C,D). Consistent with the prespecified internal-selection strategy, we instantiated RN-D3 with Spark3D as the segmentation backbone and the combined Deep+Rad logistic regression classifier as the FP-filtering post-processing step; the external results reported here were used only for final evaluation. Receiver operating characteristic (ROC) analysis of the classifier configurations confirmed this choice, with the Deep+Rad logistic-regression classifier achieving the highest area under the curve for both backbones (Spark3D 0.895; STU-Net 0.764; Supplementary Figure S2) (see Figure 4).

### 3.4 End-to-end RN-D^3^ performance

**Figure 5:**
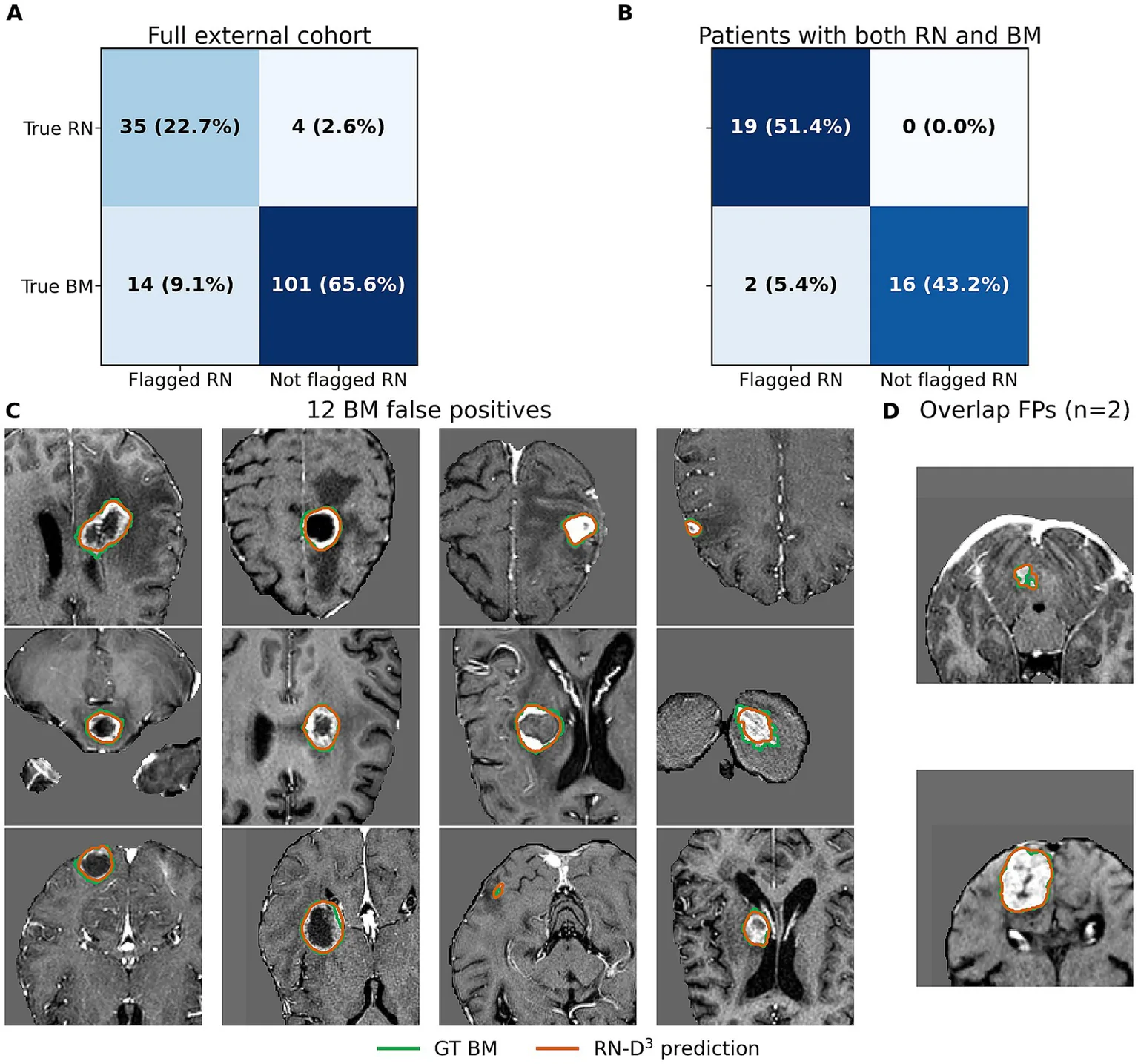
RN-D^3^ performance on RN-versus-non-RN differentiation (external MOLAB cohort). **(A)** Confusion matrix on all RN lesions, evaluated end-to-end with the 4 lesions missed at detection counted as false negatives, and all brain metastasis (BM) lesions (39 RN + 115 BM). **(B)** Confusion matrix for the subgroup of 15 patients with both RN and BM lesions (19 RN + 18 BM) All RN lesions in this subgroup were flagged by RN-D3. **(C)** The 12 BM lesions falsely flagged as RN outside the overlap subgroup, organized by morphology: Necrotic-core/ring-enhancing (column 1), solid enhancing nodules (column 2), and small or heterogeneous enhancing lesions (columns 3–4). **(D)** The 2 BM lesions falsely flagged as RN within the overlap subgroup. Post-contrast T1-weighted images show ground-truth BM contour (green) and RN-D^3^ prediction (orange).

On the full external lesion set (39 RN + 115 BM; *n* = 154), evaluated end-to-end so that the 4 RN lesions without an RN-D3 flag were counted as false negatives, RN-D3 resulted in TP = 35, FN = 4, FP = 14, and TN = 101, corresponding to sensitivity 0.897 (95% CI 0.766–0.967), specificity 0.878 (0.804–0.932), positive predictive value 0.714 (0.572–0.832), negative predictive value 0.962 (0.906–0.989), and accuracy 0.883 (0.822–0.929) (Figure 5A). In the subgroup of 15 patients with both RN and BM lesions (19 RN + 18 BM; *n* = 37), RN-D^3^ yielded TP = 19, FN = 0, FP = 2, and TN = 16, with no RN lesions missed in this subgroup (sensitivity 19/19), specificity 0.889 (0.653–0.986), and accuracy 0.946 (0.819–0.994) (Figure 5B). At the patient level, across 73 patients (29 RN-positive, 44 BM-only), and evaluated end-to-end so that patients whose only RN lesion was missed at detection were counted as false negatives, RN-D3 achieved sensitivity 86.2% (25/29; 95% CI 68.3–96.1), specificity 77.3% (34/44; 64.1–89.2), PPV 71.4% (25/35; 56.4–86.5), NPV 89.5% (34/38; 79.7–99.2), and accuracy 80.8% (59/73; 71.8–89.8). Four patients whose only RN lesion was missed at detection were counted as false negatives. Patients with both RN and BM lesions were counted as RN-positive; one BM false-positive lesion occurred in such a patient and therefore did not reduce patient-level specificity. RN-D^3^ additionally produced 8 predictions that overlapped neither with annotated RN nor BM lesions; these were not classified in the confusion matrix. To characterize residual errors, the 14 BM false positives were classified by morphology into three categories: necrotic-core/ring-enhancing lesions, solid-enhancing nodules, and small or heterogeneous enhancing lesions. Figure 5C shows the 12 BM false positives outside the overlap subgroup grouped by morphology, and Figure 5D shows the 2 BM false positives within the overlap subgroup.

## 4 Discussion

Reliable automated tools for RN detection, differentiation from progression, and delineation are increasingly needed to guide clinical decisions and enable reproducible volumetric monitoring (Ali et al., 2019; Vellayappan et al., 2018; Harrabi et al., 2022). In this study, we retrained six established CNN- and transformer-based segmentation models on a moderate-sized institutional cohort of RN, evaluated their external generalization, and combined the best-performing segmentation backbone with a false-positive classifier to form RN-D^3^. This addressed three gaps in the existing literature: the absence of external validation, the lack of evaluation of small lesions (8–10), and the lack of evaluation of RN-specific patterns versus other contrast-enhancing pathologies.

CNN-based architectures, in particular Spark3D and STU-Net, provided reliable RN segmentation on post-contrast T1-weighted MRI, achieving an external median DSC of 0.65–0.69, with complete detection of large lesions and 67–78% detection of small lesions. RN-D^3^ achieved an end-to-end sensitivity of 90 and 88% specificity against brain metastases, with missed cases attributable to detection of small lesions rather than misclassification, supporting use as a high-sensitivity triage. For small lesions (<1 mL), detection rates varied across architectures, from 67–78% for Spark3D, STU-Net, and baseline nnU-Net to 17–22% for SwinUNETR, TRIAD PlainConv, and TRIAD SwinB, identifying small lesion detection as the primary remaining challenge, where improved sensitivity would directly support earlier clinical intervention. Early initiation of RN-directed treatment has been associated with reduced all-cause mortality compared with a watch-and-wait approach, underscoring the clinical value of detecting RN at an early, small-volume stage (Pan et al., 2022). Small lesions are intrinsically harder to detect due to their size and low contrast against surrounding tissue, and are further affected by imaging variability and patient-level heterogeneity characteristic of RN (Chamseddine et al., 2024).

Among the architectures evaluated, the CNN-based models outperformed the transformer-based models under the data-limited and domain-shift conditions of this study. This is consistent with the known inductive bias of CNN encoders: local feature extraction and translation equivariance are well-suited to the small, focal, contrast-enhancing morphology of RN, whereas the transformer architectures evaluated here may require substantially larger datasets to learn meaningful global representations (Isensee et al., 2021). The controlled encoder-sharing design further revealed that the amount of pretraining alone does not determine external performance: within matched encoder groups, not all models initialized with extensive pretraining outperformed baselines trained from scratch. This observation is consistent with TRIAD’s own finding that pretraining benefits depend on task-modality alignment rather than pretraining scale alone (Yang et al., 2025). Spark3D, the largest pretrained model [90.3 M-parameter encoder, MAE pretraining on brain MRI (Wald et al., 2025)], achieved the best external performance, suggesting that large-capacity, brain-MRI-pretrained CNN encoders may be advantageous when training data are scarce, although architecture, pretraining domain, and model capacity cannot be fully disentangled in this comparison. Importantly, TRIAD was not specifically designed or optimized for RN segmentation, which differs from its pretraining domain in both lesion scale and appearance. As such, the observed performance likely reflects task mismatch rather than limitations of the model itself. The transformer models evaluated here do not represent the full range of modern or hybrid transformer and convolutional–attention architectures, and our findings should not be generalized to transformer approaches as a class.

Our external performance (median DSC: 0.65–0.69 for Spark3D and STU-Net) falls numerically below the 0.72–0.85 range reported in prior DL-based RN-related segmentation studies (8–10), but this comparison reflects two methodological differences. Prior studies reported single-institution performance (equivalent to our internal median DSC of 0.79–0.87) and exclusively addressed arteriovenous malformation- or ablation-related changes, which typically present as larger lesions than RN. When evaluated under matched single-institution conditions, our internal median DSC (0.79–0.87) overlaps the prior range, indicating that model performance is comparable when evaluation conditions are aligned.

When lesions were successfully detected, DSC was consistently higher across all models, suggesting the limiting factor is detection rather than boundary delineation. Consequently, improvements targeted at detection may be more impactful than further increases in segmentation complexity. Reliable delineation also streamlines contouring for downstream tasks such as longitudinal volumetric monitoring and mechanistic modeling of necrosis progression (Cogno et al., 2026). All models showed larger generalization gaps for small lesions, driven by the domain shift between cohorts—specifically the transition from internal thick-slice (4.0 mm) to external thin-slice (1.3 mm) MRI. This highlights a critical translational challenge: the sensitivity of deep learning models to acquisition parameters. For clinical implementation, this suggests that models trained in “thick-slice” diagnostic centers may not be “plug-and-play” for “thin-slice” stereotactic centers without institution-specific fine-tuning.

All CNN-based models produced frequent false positives in small-lesion scans, with Spark3D, nnU-Net, and STU-Net showing rates of 82, 77, and 77%, respectively. The post-processing pipeline, combining deep encoder features with shape radiomics, was applied to the two best-performing models, removing 2/13 and 7/14 FP components for Spark3D and STU-Net, respectively, while retaining all true detections for Spark3D and 85% for STU-Net. Shape radiomics alone preserved all true detections but did not distinguish true from false positives, whereas deep features did so at the cost of removing some true positives; the combination was required to achieve both sensitivity preservation and FP reduction. However, robust FP suppression without sensitivity loss remains an open problem.

Combining the best detection/segmentation backbone (Spark3D) with the FP classifier yielded RN-D3, an end-to-end pipeline that addresses detection, differentiation, and delineation. In the external cohort, RN-D^3^ achieved 90% end-to-end sensitivity and 88% specificity for brain metastases, including 89% specificity in the predefined subgroup of patients with both pathologies, in which all RN lesions were correctly identified. The 14 BM lesions falsely flagged as RN included necrotic-core/ring-enhancing morphology, solid enhancing nodules, and small or heterogeneous enhancing lesions, features that overlap visually with RN, suggesting that residual errors reflect inherent RN-versus-progression diagnostic challenges rather than model-specific artifacts. In clinical workflow, this profile would allow RN-D3 to flag candidate RN lesions for confirmation while providing high-confidence reassurance for unflagged regions.

This study aimed not to optimize a single model, but to evaluate what can be achieved with existing approaches on real-world clinical data. Several limitations should be noted. The training cohort is small (*n* = 52) and from a single institution, reflecting typical data availability in many clinical settings. This data-scarce regime is precisely the setting in which pretrained and foundation-model initialization is expected to be advantageous and motivated our evaluation of these strategies alongside extensive regularization and per-architecture hyperparameter tuning to limit overfitting. At this scale, however, the study remains underpowered for definitive architecture comparison: the leading models were statistically indistinguishable, and their ordering should be read as a ranking under data-limited conditions rather than as evidence of true superiority. The two cohorts differed in both RT modality (fractionated proton beam internally versus photon-based SRS, with or without WBRT, externally) and MRI acquisition protocol (median slice thickness 4.0 mm vs. 1.3 mm), representing confounders that may have contributed to the observed generalization gap. Preprocessing followed the pretrained models’ published specifications; sequence-specific intensity normalization may further improve cross-scanner generalization (Salome et al., 2023b). SRS may produce radiographic changes with different temporal patterns than fractionated RT. RN diagnosis relied on imaging-based criteria rather than systematic histopathological confirmation; histopathological correlation in future studies could further strengthen diagnostic certainty. Inter-observer agreement of RN annotation was not formally quantified. Although the cohorts differ in pathology, modality, and site, the entity under study, radiation-induced injury to normal brain tissue, shares a common pathophysiology and post-contrast imaging appearance independent of the underlying malignancy or delivery technique (Shah et al., 2012), and the consistent external CNN performance supports the learnability of this shared phenotype. Skull-base lesions may nonetheless differ in appearance from parenchymal radionecrosis, warranting confirmation in site-specific cohorts. Our external specificity evaluation compares RN against brain-metastasis lesions rather than recurrent or progressive tumor at the treated site, and therefore assesses discrimination from other enhancing pathologies rather than the clinical RN-versus-recurrence decision. Because RN-D^3^ is intended to characterize lesions in which RN is already clinically suspected rather than to screen unselected patients, the study population, selected for radiographically identified RN, reflects this intended use; nonetheless, the cohort comprises lesions with an established RN or brain-metastasis determination and may not fully represent lesions encountered in routine surveillance, so performance may not transfer directly to unselected clinical practice.

All models were trained on post-contrast T1-weighted MRI only, as the MOLAB dataset does not include additional sequences; incorporating multiparametric MRI, radiological reports, radiation dose maps, or prompt-based segmentation [e.g., MedSAM (Ma et al., 2024b)] may improve small-lesion sensitivity and reduce false positives. Evaluation on larger multi-institutional cohorts remains an important next step.

## 5 Conclusion

We present RN-D^3^, an end-to-end pipeline that integrates a CNN segmentation backbone with an RN-versus-non-RN classifier for the detection, differentiation, and delineation of radiation necrosis. On external data, RN-D^3^ achieved high end-to-end sensitivity and high specificity against brain metastases, with small-lesion detection the primary factor limiting sensitivity. Across heterogeneous proton and photon radiosurgery cohorts, the CNN-based architectures evaluated provided the most reliable segmentation, with small-lesion detection remaining the primary translational bottleneck.

## Data Availability

All code and trained model weights are publicly available at: https://github.com/chamseddine-lab/rn-d3. Due to patient privacy concerns and institutional review board restrictions, individual patient-level data, including MRI imaging files, cannot be made publicly available. The external MOLAB dataset is openly accessible under a Creative Commons Attribution 4.0 license at: https://doi.org/10.6084/m9.figshare.c.6194104.v1. Deidentified aggregate data from the internal cohort may be available from the corresponding author upon reasonable request and with appropriate institutional agreements.

## References

[ref1] Ali F. S. Arevalo O. Zorofchian S. Patrizz A. Riascos R. Tandon N. . (2019). Cerebral radiation necrosis: incidence, pathogenesis, diagnostic challenges, and future opportunities. Curr. Oncol. Rep. 21:66. doi: 10.1007/s11912-019-0818-y, 31218455

[ref2] Cao Y. Parekh V. S. Lee E. Chen X. Redmond K. J. Pillai J. J. . (2023). A multidimensional Connectomics- and Radiomics-based advanced machine-learning framework to distinguish radiation necrosis from true progression in brain metastases. Cancers (Basel) 15:4113. doi: 10.3390/cancers15164113, 37627141 PMC10452423

[ref3] Chamseddine I. Shah K. Lee H. Ehret F. Schuemann J. Bertolet A. . (2024). Decoding patient heterogeneity influencing radiation-induced brain necrosis. Clin. Cancer Res. 30, 4424–4433. doi: 10.1158/1078-0432.CCR-24-1215, 39106090 PMC11444871

[ref4] Cogno N. Shah K. D. Ehret F. Beekman C. Enderling H. Dawson R. J. . (2026). A mechanistic model of brain necrosis progression based on vascular heterogeneity. Int. J. Radiat. Oncol. Biol. Phys. 124, 842–855. doi: 10.1016/j.ijrobp.2025.09.059, 41083028 PMC12688031

[ref5] Harrabi S. B. von Nettelbladt B. Gudden C. Adeberg S. Seidensaal K. Bauer J. . (2022). Radiation induced contrast enhancement after proton beam therapy in patients with low grade glioma—how safe are protons? Radiother. Oncol. 167, 211–218. doi: 10.1016/j.radonc.2021.12.035, 34973277

[ref6] Haskell-Mendoza A. P. Reason E. H. Gonzalez A. T. Jackson J. D. Sankey E. W. Srinivasan E. S. . (2024). Automated segmentation of ablated lesions using deep convolutional neural networks: a basis for response assessment following laser interstitial thermal therapy. Neuro-Oncology 26, 1152–1162. doi: 10.1093/neuonc/noad261, 38170451 PMC11145442

[ref7] Hatamizadeh A. Nath V. Tang Y. Yang D. Roth H. Xu D. (2022). Swin UNETR: swin transformers for semantic segmentation of brain tumors in MRI images. arXiv. doi: 10.48550/arXiv.2201.01266

[ref8] Ho H. H. Yang H. C. Yang W. X. Lee C. C. Wu H. M. Lai I. C. . (2025). Deep learning for automated segmentation of radiation-induced changes in cerebral arteriovenous malformations following radiosurgery. BMC Med. Imaging 25:218. doi: 10.1186/s12880-025-01796-w, 40597846 PMC12210579

[ref9] Huang Z. Wang H. Deng Z. Ye J. Su Y. Sun H. . (2023). STU-net: scalable and transferable medical image segmentation models empowered by large-scale supervised pre-training. arXiv. doi: 10.48550/arXiv.2304.06716

[ref10] Isensee F. Jaeger P. F. Kohl S. A. A. Petersen J. Maier-Hein K. H. (2021). nnU-net: a self-configuring method for deep learning-based biomedical image segmentation. Nat. Methods 18, 203–211. doi: 10.1038/s41592-020-01008-z, 33288961

[ref11] Isensee F. Schell M. Pflueger I. Brugnara G. Bonekamp D. Neuberger U. . (2019). Automated brain extraction of multisequence MRI using artificial neural networks. Hum. Brain Mapp. 40, 4952–4964. doi: 10.1002/hbm.24750, 31403237 PMC6865732

[ref12] Lawrence Y. R. Li X. A. el Naqa I. Hahn C. A. Marks L. B. Merchant T. E. . (2010). Radiation dose-volume effects in the brain. Int. J. Radiat. Oncol. Biol. Phys. 76, S20–S27. doi: 10.1016/j.ijrobp.2009.02.091, 20171513 PMC3554255

[ref13] Ma J. He Y. Li F. Han L. You C. Wang B. (2024a). Segment anything in medical images. Nat. Commun. 15:654. doi: 10.1038/s41467-024-44824-z, 38253604 PMC10803759

[ref14] Ma J. He Y. Li F. Han L. You C. Wang B. (2024b). Segment anything in medical images. Nat. Commun. 15:654. doi: 10.1038/s41467-024-44824-z, 38253604 PMC10803759

[ref15] Niemierko A. Schuemann J. Niyazi M. Giantsoudi D. Maquilan G. Shih H. A. . (2021). Brain necrosis in adult patients after proton therapy: is there evidence for dependency on linear energy transfer? Int. J. Radiat. Oncol. Biol. Phys. 109, 109–119. doi: 10.1016/j.ijrobp.2020.08.058, 32911019 PMC7736370

[ref16] Niyazi M. Niemierko A. Paganetti H. Söhn M. Schapira E. Goldberg S. . (2020). Volumetric and actuarial analysis of brain necrosis in proton therapy using a novel mixture cure model. Radiother. Oncol. 142, 154–161. doi: 10.1016/j.radonc.2019.09.008, 31563411

[ref17] Ocaña-Tienda B. Pérez-Beteta J. Villanueva-García J. D. Romero-Rosales J. A. Molina-García D. Suter Y. . (2023). A comprehensive dataset of annotated brain metastasis MR images with clinical and radiomic data. Sci. Data 10:208. doi: 10.1038/s41597-023-02123-0, 37059722 PMC10104872

[ref18] Pan D. Rong X. Chen D. Jiang J. Ng W. T. Mai H. . (2022). Mortality of early treatment for radiation-induced brain necrosis in head and neck cancer survivors: a multicentre, retrospective, registry-based cohort study. EClinicalMedicine 52:101618. doi: 10.1016/j.eclinm.2022.101618, 36034411 PMC9399256

[ref19] Ressa G. Levi R. Savini G. Raspagliesi L. Clerici E. Bellu L. . (2025). AI differentiates radionecrosis from true progression in brain metastasis upon stereotactic radiosurgery: analysis of 124 histologically assessed lesions. Neuro-Oncology 27, 3029–3039. doi: 10.1093/neuonc/noaf090, 40172476 PMC12908478

[ref20] Ronneberger O. Fischer P. Brox T. (2015). U-net: convolutional networks for biomedical image segmentation. arXiv. doi: 10.48550/arXiv.1505.04597

[ref21] Sadique M. S. Farzana W. Temtam A. Lappinen E. Vossough A. Iftekharuddin K. M. (2024). Brain tumor recurrence vs. radiation necrosis classification and patient survivability prediction. IEEE J. Biomed. Health Inform. 28, 5685–5695. doi: 10.1109/JBHI.2024.3406256, 38875078 PMC11492206

[ref22] Salari E. Elsamaloty H. Ray A. Hadziahmetovic M. Parsai E. I. (2023). Differentiating radiation necrosis and metastatic progression in brain tumors using Radiomics and machine learning. Am. J. Clin. Oncol. 46, 486–495. doi: 10.1097/COC.0000000000001036, 37580873 PMC10589425

[ref23] Salome P. Sforazzini F. Brugnara G. Kudak A. Dostal M. Herold-Mende C. . (2023a). MR-class: a Python tool for brain MR image classification utilizing one-vs-all DCNNs to Deal with the open-set recognition problem. Cancer 15. doi: 10.3390/cancers15061820, 36980707 PMC10046648

[ref24] Salome P. Sforazzini F. Brugnara G. Kudak A. Dostal M. Herold-Mende C. . (2023b). MR intensity normalization methods impact sequence specific Radiomics prognostic model performance in primary and recurrent high-grade glioma. Cancer 15:965. doi: 10.3390/cancers15030965, 36765922 PMC9913466

[ref25] Shah R. Vattoth S. Jacob R. Manzil F. F. P. O’Malley J. P. Borghei P. . (2012). Radiation necrosis in the brain: imaging features and differentiation from tumor recurrence. Radiographics 32, 1343–1359. doi: 10.1148/rg.325125002 22977022

[ref26] van Griethuysen J. J. M. Fedorov A. Parmar C. Hosny A. Aucoin N. Narayan V. . (2017). Computational Radiomics system to decode the radiographic phenotype. Cancer Res. 77, e104–e107. doi: 10.1158/0008-5472.CAN-17-0339, 29092951 PMC5672828

[ref27] Vellayappan B. Tan C. L. Yong C. Khor L. K. Koh W. Y. Yeo T. T. . (2018). Diagnosis and Management of Radiation Necrosis in patients with brain metastases. Front. Oncol. 8:395. doi: 10.3389/fonc.2018.00395, 30324090 PMC6172328

[ref28] Wald T. Ulrich C. Lukyanenko S. Goncharov A. Paderno A. Miller M. . (2025). Revisiting MAE pre-training for 3D medical image segmentation. arXiv. doi: 10.48550/arXiv.2410.23132

[ref29] Wasserthal J. Breit H. C. Meyer M. T. Pradella M. Hinck D. Sauter A. W. . (2023). TotalSegmentator: robust segmentation of 104 anatomic structures in CT images. Radiology 5:e230024. doi: 10.1148/ryai.230024, 37795137 PMC10546353

[ref30] Yang X. Wang S. Safari M. Li Q. Chang C. W. Qiu R. Triad: Vision Foundation Model for 3D Magnetic Resonance Imaging Research Square (2025) (Accessed April 7, 2026). Available online at: https://www.researchsquare.com/article/rs-6129856/v1

[ref31] Zhou Z. Sodha V. Pang J. Gotway M. B. Liang J. (2021). Models Genesis. Med. Image Anal. 67:101840. doi: 10.1016/j.media.2020.101840, 33188996 PMC7726094

