# Supplementary material for: RN-D3: detection, differentiation, and delineation of radiation necrosis on post-contrast MRI
Source: Front Artif Intell. 2026 Aug 6;9:1891883. doi: 10.3389/frai.2026.1891883 (PMC13490108; doi:10.3389/frai.2026.1891883)
Supplement: Supplementary file 1 [file Data_Sheet_1.DOCX]

Supplementary material

**Supplementary table S1.** Patient and imaging characteristics. Values are median (IQR) or n (%) unless stated otherwise. P-values from Mann–Whitney U (continuous) or Fisher’s exact test (categorical). RBE = relative biological effectiveness; SRS = stereotactic radiosurgery; WBRT = whole-brain radiotherapy.

| **Characteristic** | **Internal cohort (n = 52)** | **External cohort (MOLAB, n = 29)** |
| --- | --- | --- |
| Age, years | 53.2 (45.8–63.6) | 59.0 (51.0–65.0) |
| Sex, M | 29 (56%) | 15 (52%) |
| Sex, F | 23 (44%) | 14 (48%) |
| RT modality |  |  |
| Protons | 52 (100%) | — |
| SRS | — | 20 (69%) |
| SRS + WBRT | — | 9 (31%) |
| Dose | 68.3 Gy(RBE)  (59.4–80.5) | SRS: 20.0 (16.0–24.0); SRS+WBRT: 30.0 (16.5–75.0) |
| Fractions | 35 (30–40) | SRS: 1; SRS+WBRT: 3–15 |
| Time to RN, months median (IQR) | 18 (12–31.5) | 14.0 (10.4–19.3) |
| Collection period | 2004–2016 | 2005–2021 |
| Slice thickness, mm median (range) | 4.0 (1.0–5.0) | 1.3 (1.0–2.0) |
| Scanner manufacturers | Siemens, Philips, GE | Siemens, Philips, GE |
| Unique scanner models | 13 | 5 |
| Volume <1 mL, n (%) | 21 (40%) | 18 (46%) |
| Volume ≥1 mL, n (%) | 31 (60%) | 21 (54%) |

**Supplementary figure S1.** Training dynamics across five CV folds for all 6 models. (A) Validation DSC per epoch. (B) Training loss per epoch. Shaded bands: ±1 SD across folds.

**Supplementary table S2.** Architecture and pretraining details. MAE = masked autoencoder; SSL = self-supervised learning; FM = foundation model.

| **Model** | **Backbone** | **Total params (M)** | **Encoder params (M)** | **Encoder channels** | **Pretrainig**  **approach** | **Pretraining dataset** |
| --- | --- | --- | --- | --- | --- | --- |
| nnU-Net | CNN (ResEnc) | 30.4 | 14.0 | 32→64→128→256→320→320 | None (from scratch) | — |
| STU-Net | CNN (ResEnc) | 30.4 | 14.0 | 32→64→128→256→320→320 | Supervised | TotalSegmentator (>100k CT) |
| SwinUNETR | Transformer (Swin-B) | 39.8 | 18.6 | 48→96→192→384→768 | SSL (MAE) | BraTS + body MRI |
| TRIAD PlainConv | CNN (ResEnc) | 30.4 | 14.0 | 32→64→128→256→320→320 | Foundation model | TriadMR-131K (131,170 MRI) |
| TRIAD SwinB | Transformer (Swin-B) | 39.8 | 18.6 | 48→96→192→384→768 | Foundation model | TriadMR-131K (131,170 MRI) |
| Spark3D | CNN (ResEnc-L) | 106.7 | 90.3 | 32→64→128→256→320→320 | SSL (MAE) | 39,000 brain MRI |

**Supplementary table S3.** Best hyperparameter configurations from per-model grid search.

| **Model** | **Learning rate** | **Weight decay** | **Encoder LR multiplier** | **Freeze epochs** |
| --- | --- | --- | --- | --- |
| nnU-Net (baseline) | 2.0 × 10⁻⁴ | 1.0 × 10⁻⁵ | 1.0 | 0 |
| STU-Net | 1.0 × 10⁻⁴ | 1.0 × 10⁻⁴ | 0.1 | 10 |
| SwinUNETR | 5.0 × 10⁻⁵ | 1.0 × 10⁻⁴ | 0.1 | 5 |
| TRIAD PlainConv | 1.0 × 10⁻⁴ | 1.0 × 10⁻⁵ | 0.1 | 10 |
| TRIAD SwinB | 5.0 × 10⁻⁵ | 1.0 × 10⁻⁴ | 0.1 | 5 |
| Spark3D | 3.0 × 10⁻⁴ | 1.0 × 10⁻⁵ | 0.1 | 5 |

**Supplementary figure S2.** ROC curves for four classifier configurations—deep features (Deep only), shape radiomics (Rad only), and their combination (Deep+Rad), each with logistic regression (LR), plus Deep+Rad with XGBoost (XGB)—for the Spark3D (left) and STU-Net (right) backbones, computed over the external candidates (39 RN lesions plus 13 Spark3D or 14 STU-Net false positives). The Deep+Rad LR classifier achieved the highest AUC for both backbones (Spark3D 0.895; STU-Net 0.764) and was selected for RN-D3.
